# Continuous evolution of clinical phenotype in 578 Japanese patients with Behçet’s disease: a retrospective observational study

**DOI:** 10.1186/s13075-016-1115-x

**Published:** 2016-10-03

**Authors:** Yohei Kirino, Haruko Ideguchi, Mitsuhiro Takeno, Akiko Suda, Kana Higashitani, Yosuke Kunishita, Kaoru Takase-Minegishi, Maasa Tamura, Toshiyuki Watanabe, Yukiko Asami, Takeaki Uehara, Ryusuke Yoshimi, Tetsu Yamazaki, Akiko Sekiguchi, Atsushi Ihata, Shigeru Ohno, Atsuhisa Ueda, Toshihisa Igarashi, Shohei Nagaoka, Yoshiaki Ishigatsubo, Hideaki Nakajima

**Affiliations:** 1 Department of Stem Cell and Immune Regulation, Yokohama City University Graduate School of Medicine, 3-9 Fukuura, Kanazawa-Ku, Yokohama, 236-0004 Japan; 2 Department of Rheumatology, Yokohama Minami Kyosai Hospital, Yokohama, Japan; 3 Department of Allergy and Rheumatology, Nippon Medical School Graduate School of Medicine, Tokyo, Japan; 4 Yokosuka Center for Rheumatic Diseases, Yokosuka City Hospital, Yokosuka, Japan; 5 Center for Rheumatic Diseases, Yokohama City University Medical Center, Yokohama, Japan; 6 Department of Rheumatology, National Hospital Organization Yokohama Medical Center, Yokohama, Japan; 7 Department of Rheumatology, Chigasaki City Hospital, Chigasaki, Japan; 8 Department of Rheumatology, Yamato City Hospital, Yamato, Japan; 9 Department of Hematology and Rheumatology, Fujisawa City Hospital, Fujisawa, Japan; 10 Yokohama City University, Yokohama, Japan; 11 Y-CURD study group, Yokohama, Japan

**Keywords:** Behçet’s disease, Evolution, HLA-B51, Complete type

## Abstract

**Background:**

It has been suggested that the phenotypes of Behçet’s disease (BD) in Japan are changing. To ask whether the evolution of BD holds true in recent-onset cases in Japan, we performed a retrospective study.

**Methods:**

We reviewed the records of 578 patients with BD who met the 1987 revised diagnostic criteria of the Behçet’s disease research committee of Japan. The patients were divided into three groups based on the date of disease onset. We compared the demography, clinical features, and treatments among them with or without adjustment for the observation period. Patients having oral ulcers, genital ulcers, regional skin involvement, and uveitis are categorized as having complete-type BD, and the associated factors were determined by univariate and multivariate logistic regression analyses.

**Results:**

Male patients had a higher propensity for uveitis and central nervous system (CNS) involvement, whereas female patients had higher rates of genital ulcers and arthritis. We found a significant trend in reduction of complete-type, genital ulcer, HLA-B51 carriers, and increment of gastrointestinal BD over time. Multiple regression analysis identified HLA-B51 positivity, earlier date of disease onset, and younger age of onset as independently associated with complete-type BD. Although treatments had been also chronologically changed, the causative relationship between therapeutic agents and phenotypical changes was not determined from the study.

**Conclusion:**

The present study revealed that phenotypical evolution was characterized by decreased incidence of the complete type and increment of gastrointestinal involvement in Japanese patients with BD during the last 30 years.

**Electronic supplementary material:**

The online version of this article (doi:10.1186/s13075-016-1115-x) contains supplementary material, which is available to authorized users.

## Background

Behçet’s disease (BD) is an inflammatory disease of unknown cause, affecting multiple organs such as eyes, skin, mucosa, and brain [1]. A definitive environmental factor associated with BD is still unknown. Microbes such as *Streptococcus sanguinis* and herpes simplex have been implicated in BD pathogenesis [2, 3], and genetic evidence supporting association with microbiomes in the development of BD is growing [4]. Moreover, dysbiosis in the intestinal flora has been reported in many inflammatory diseases including BD [5, 6]. It is thus possible that environmental factors modify the clinical courses and phenotypes of BD.

Epidemiological studies from Japan and Korea have shown that the prevalence of BD, particularly of patients with serious manifestations, is decreasing [7–9]. We previously reported that newly diagnosed complete-type BD (patients having all four of the major symptoms of oral ulcers, genital ulcers, and eye and skin lesions) in Kanagawa district, Japan had been declining since the year 2000, compared with the previous data (33 % of patients diagnosed before 2000 had complete BD compared to 23 % of patients between 2000 and 2007) [8]. Similarly, a recent paper from Korea also reported reduction in the complete type, declining male propensity, and shifting patterns of organ involvement, during the last 30 years [9]. However, to our knowledge, such chronological changes in the BD phenotype have been reported only in Japan and Korea, where the majority of the residents have been native and genetically homogenous, with infrequent influx of immigrants from other ethnic groups. These findings suggest the involvement of altered environmental factors in the phenotypical changes in BD in these countries. To determine whether the trends were transient or continuous in the same area, this study enrolled patients newly diagnosed with BD after 2008 and compared them with the previous cohorts reported by Ideguchi et al [8].

## Methods

The patients who participated in the study met the 1987 revised diagnostic criteria of the Behçet’s disease research committee, the Ministry of Health, Labor and Welfare of Japan [10]. These patients had been treated in any of the seven hospitals within the Kanagawa district located in mid-Japan, from July 1991 to December 2015. All patients were Japanese except for four patients (one Filipino, one Syrian, one Korean, and one Chinese patient). The study was approved by the Ethic Committee of Yokohama City University (A141127010). The study followed the Ethical Guidelines for Epidemiology Research, published by the Japanese Ministry of Health, Labor, and Welfare, applying the opt-out strategy. In addition to patients who participated in the study reported by Ideguchi, we extracted all of the patients with a diagnosis of BD from the computerized medical records in the seven participating hospitals. Thereafter, different rheumatologists (YK Kirino, HK, AS Suda, Y Kunishita, KM, TM, TW, YA, TU, RY, TY, and AS Sekiguchi) reviewed the records for their clinical manifestations and treatment regimens.

Under the Japanese criteria, recurrent aphthous oral ulcers, skin lesions, ocular inflammation, and genital ulcers are included in “major symptoms”, whereas arthritis, intestinal ulcers, epididymitis, vascular lesions, and neuropsychiatric disease are included under “minor symptoms”. The criteria classify clinical subtypes into complete and incomplete types. The former includes patients with all four major symptoms during the clinical course, whereas the latter includes those having three major symptoms, two major and wo minor symptoms, typical recurrent ocular inflammation and one or more major symptoms, or typical recurrent ocular inflammation and two minor symptoms. Moreover, patients with central nervous system (CNS) involvement, vascular, and gastrointestinal (GI) region involvement were categorized as having special-type BD, and were further defined as the neurologic type, vascular type, and GI type, respectively.

To reduce the heterogeneity of patients with BD and to be comparable with other BD studies, we also applied International Study Group (ISG) criteria to our patients [11]. We extracted the following variables from the patients’ records; BD phenotype, sex, age of BD onset, date of disease onset, HLA-B51 positivity, observation period, and treatment with biologic agents.

### Statistical analysis

Statistical analysis was performed with GraphPad Prism 6 (San Diego, CA, USA) and SPSS version 22 (IBM Japan, Tokyo, Japan). Categorical variables were analyzed using the chi-square test or Cochran–Armitage test. Continuous variables were analyzed using the Student *t* test. Univariate and forward stepwise multivariate logistic regression analysis was performed with presence of complete-type BD as the dependent variable. Sex and age of BD onset were forced into into the model. Variables that were significant in univariate analysis were further included in the multivariate analysis. A *p* value less than 0.05 was considered as statistically significant.

## Results

**Fig. 1:**
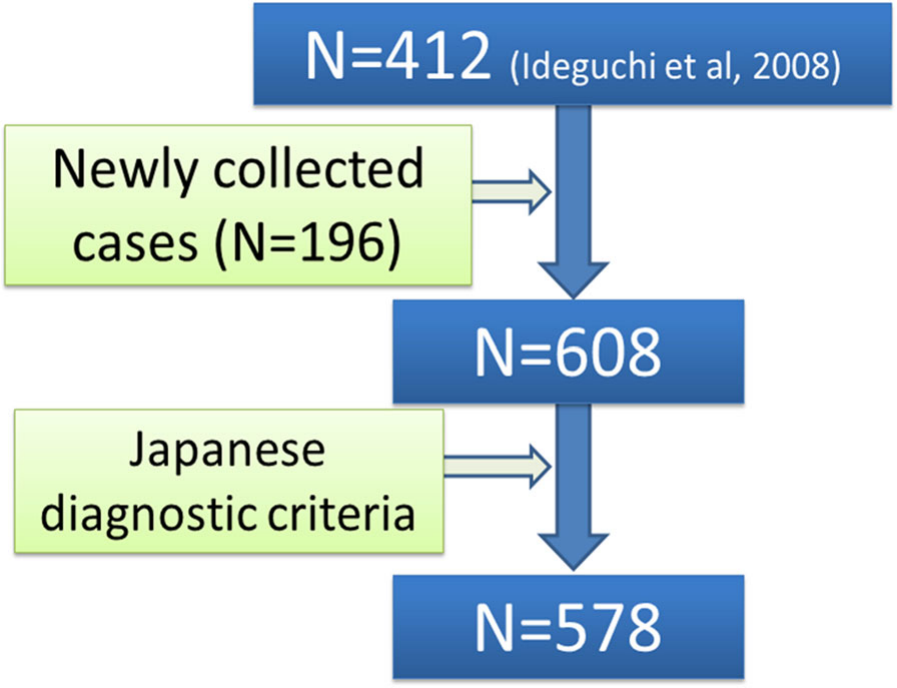
Scheme of the patients with Behçet’s disease who participated in the study

We first carefully examined patients’ records and identified 578 patients who met the 1987 revised Japanese BD diagnostic criteria, including 412 patients reported in the previous paper [8] (Fig. 1). The clinical characteristics are shown in Table 1. Consistent with our previous data [8], genital ulcers and arthritis were more frequent in female patients, whereas eye disease and CNS involvement were more common in male patients. There was no difference in HLA-B51 positivity between male and female patients: both had higher HLA-B51 positivity than the general Japanese population (about 15 %), indicating that the estimated odds ratio of HLA-B51 positivity in BD was approximately 3.5 in this series.

**Table 1:** Characteristic of patients with Behçet’s disease (BD) who were enrolled in the study

Characteristics	All (n = 578)	Male (n = 247)	Female (n = 331)	*P* ^a^
Age at onset (mean ± SD)	36.8 ± 12.4	35.2 ± 12.1	37.8 ± 12.0	**0.013** ^**b**^
Observation period (mean ± SD)	9.2 ± 9.2	8.2 ± 7.6	9.6 ± 8.4	**0.036** ^**b**^
HLA-B51, *n* (%)	177/352 (50.3)	88/170 (51.8)	89/182 (48.9)	0.59
Oral ulcer, *n* (%)	572 (99.0)	246 (99.6)	326 (98.5)	0.11
Genital ulcer, *n* (%)	417 (72.3)	141 (57.3)	276 (83.4)	**<0.0001**
Eye involvement, *n* (%)	356 (61.6)	181 (73.3)	175 (52.9)	**<0.0001**
Skin involvement, *n* (%)	513 (88.8)	212 (85.8)	301 (90.9)	0.055
Arthritis, *n* (%)	301 (52.1)	104 (42.1)	197 (59.5)	**<0.0001**
Epididymitis, *n* (%)	14 (2.4)	14 (5.7)		
Gastrointestinal involvement, *n* (%)	71(12.3)	26 (10.5)	45 (13.6)	0.069
CNS involvement, *n* (%)	59 (10.2)	34 (13.8)	25 (7.6)	**0.015**
Vascular involvement, *n* (%)	46 (8.0)	25 (10.1)	21 (6.3)	0.097
Fulfilling ISG, *n* (%)	517 (89.4)	213 (86.2)	304 (91.8)	**0.030**

^a^Male vs. female groups. ^b^Analyzed using the unpaired *t* test. Other variables were analyzed using the chi-square test. *CNS* central nervous system, *ISG* International Study Group criteria for BD. Significant results are highlighted in bold font

As mentioned earlier, we had divided the patients into two groups of patients diagnosed before 2000 and those diagnosed between 2000 and 2007, in the previous paper [8]. Therefore, in the current study, the patients were divided into three groups; group A (before year 2000), group B (between 2000 and 2007), and group C (after 2008, after the publication by Ideguchi), according to the date of BD diagnosis (Table 2). In the previous study there were significant reductions in the frequency of the complete type, of genital ulcers, skin involvement, and CNS involvement in group B compared to group A [8]. As shown in Table 2 we confirmed that these trends were continuous after 2008. However, there was also significant differences in the observation period among the three groups.

**Table 2:** Evolution of clinical phenotypes of Behçet’s disease in Japan before correction for the follow-up period

	Group A pre 2000 (n = 323)	Group B 2000–2007 (n = 164)	Group C Post 2008 (n = 91)	*P*
Observation time (years)	11.9 ± 9.3	6.3 ± 4.8	3.7 ± 2.2	**<0.0001**
Age at onset	36.9 ± 11.8	35.8 ± 13.1	37.6 ± 11.4	NS
Gender male, *n* (%)	134 (41.5)	76 (46.3)	37 (40.7)	0.80
HLA-B51, *n* (%)	107 (54.9)	48 (49.0)	16 (43.1)	0.061^a^
Complete type, *n* (%)	124 (38.4)	45 (27.4)	16 (17.6)	**<0.0001**
Oral ulcer, *n* (%)	321 (99.4)	163 (99.4)	88 (96.7)	0.065
Genital ulcer, *n* (%)	246 (76.2)	116 (70.7)	55 (60.4)	**0.0033**
Eye involvement, *n* (%)	211 (65.3)	92 (56.1)	53 (58.2)	0.083
Skin involvement, *n* (%)	293 (90.7)	145 (88.4)	75 (82.4)	**0.033**
Arthritis, *n* (%)	166 (51.4)	87 (53.0)	48 (52.7)	0.75
Gastrointestinal involvement, *n* (%)	37 (11.5)	20 (12.2)	14 (16.5)	0.35
CNS involvement, *n* (%)	39 (12.1)	17 (10.4)	3 (3.3)	**0.023**
Vascular involvement, *n* (%)	26 (8.1)	17 (10.4)	3 (3.3)	0.35

^a^Not all of the patients were typed for HLA-B51 positivity. Significant results are highlighted in bold font. *CNS* central nervous system, *NS* not significant

In our previous study the clinical picture was of BD evolving even after achieving the definitive diagnosis and of patients with a longer history of BD presenting with more manifestations [8]. Thus, we next adjusted the observation duration among the three groups by selecting symptoms appearing within 4.5 years after the onset of disease. Patients without data on the onset of individual manifestations were excluded from the analysis (Table 3). Even after the adjustment, the date-dependent reduction in the complete-type BD and genital ulcers remained statistically significant. In addition, there was a reduced rate of HLA-B51 positivity and increased rate of the GI type. There were no significant shifts in the rate of male patients or age of disease onset, which are predisposing factors to the severe BD phenotype [12, 13]. Although the frequency of genital ulcers was significantly reduced in female patients (*p* = 0.025 compared p = 0.13 for male patients), this symptom was still more common in female than in male patients.

**Table 3:** Evolution of clinical phenotypes of Behçet’s disease in Japan after correction for the follow-up period

	Group A Pre 2000 (n = 293)	Group B 2000–2007 (n = 160)	Group C Post 2008 (n = 91)	*P*
Observation time (years)	3.52 ± 1.6	3.25 ± 1.7	3.7 ± 2.2	NS
Age at onset	36.4 ± 11.9	35.8 ± 13.0	37.6 ± 11.4	NS
Gender male, *n* (%)	121 (41.3)	74 (46.3)	37 (40.7)	0.81
HLA-B51, *n* (%)	103 (53.4)	47 (50.0)	16 (43.1)	**0.031** ^a^
Complete type, *n* (%)	99 (33.8)	37 (23.1)	16 (17.6)	**0.0008**
Oral ulcer, *n* (%)	291 (99.3)	159 (99.4)	88 (96.7)	0.079
Genital ulcer, *n* (%)	219 (74.7)	112 (70.0)	55 (60.4)	**0.0096**
Eye involvement, *n* (%)	184 (62.8)	85 (53.1)	53 (58.2)	0.19
Skin involvement, *n* (%)	255 (87.0)	137 (85.6)	75 (82.4)	0.28
GI, *n* (%)	18 (6.1)	19 (11.9)	14 (16.5)	**0.0036**
CNS, *n* (%)	20 (6.8)	14 (8.8)	3 (3.3)	0.46
Vascular, *n* (%)	17 (5.8)	15 (9.4)	3 (3.3)	0.73
Complete type fulfilling, *n* ISG (%)	99/264 (37.5)	37/135 (27.4)	16/74 (21.6)	**0.0037**
Complete type w/o GI, *n* (%)	96/275 (34.9)	35/141 (24.8)	15/77 (19.5)	**0.0030**

Numbers of patients shown are different from Table 2 because 31 patients were excluded due to ambiguity of the date of phenotype expression. ^a^Not all of the patients were typed for HLA-B51 positivity. *GI* gastrointestinal, *CNS* central nervous system, *w/o* without, *NS* not significant

All patients met the Japanese criteria in this study, but 61 patients (10.6 %) did not meet the ISG criteria (Table 1). On the other hand, patients fulfilling the ISG criteria but not the Japanese criteria were not enrolled found in our cohort, because the ISG criteria list only four symptoms besides a positive pathergy test, and phenotypical diversity is more restricted than in the Japanese criteria. The frequency of complete-type BD was chronologically reduced or limited in patients fulfilling the ISG criteria (Table 3).

The frequency of patients with the GI type of BD have been recently increasing. We and others have shown that these patients have a lower frequency of HLA-B51 positivity, complete-type disease, and uveitis [14, 15], suggesting that the GI type might have unique genetic and phenotypical features in comparison with patients who have other subtypes of BD [16] (Additional file 1: Table S1). To examine whether recruitment of more patients with GI type BD in group C could result in a reduction in the rate of complete-type BD, we looked at the rates of the complete type in patients without GI involvement. Even after excluding these patients, there was a significant reduction in the complete type (Table 3).

A meta-analysis has shown that HLA-B51 is associated with male predominance and moderately higher prevalence of genital ulcers, skin involvement, ocular involvement, and decreased prevalence of GI involvement [14]. Therefore, a reduced rate of HLA-B51-positive patients in our cohort could be implicated in the increased frequency of the milder disease phenotype. However, our data revealed that the phenotypical evolution was found in HLA-B51-positive patients as well as in other patients (the rate of the complete type in HLA-B51-positive cases was 46.6 % before 2000, 29.8 % in 2000–2007, and 31.8 % in 2008–2015), though the sample size was presumably too small (n = 172) to obtain a statistically significant result (*p* = 0.062).

In the current study, patients were arbitrarily segmented into three groups based on disease onset, which may have resulted in skewed outcomes. In addition, multiple factors mutually contribute to the reduced rates of complete-type BD. To identify factors independently associated with complete-type BD, we performed univariate and multivariate logistic regression analyses, with the complete type as a dependent variable. In multivariate analysis, young age of onset, HLA-B51 positivity, and earlier date of diagnosis were independently associated with risk of developing complete-type BD (Table 4).

**Table 4:** Univariate and multivariate logistic regression analysis with the complete type as a dependent variable

	Univariate				Multivariate			
	*P*	Odds ratio	95 % CI		*P*	Odds ratio	95 % CI	
Age of onset	**0.024**	0.98	0.97	1.00	**0.049**	0.98	0.96	1.00
Sex	0.275	0.82	0.58	1.17	0.26	0.77	0.49	1.22
Date of onset	**1.1 × 10** ^**-5**^	0.37	0.24	0.58	**0.02**	0.50	0.28	0.90
Gastrointestinal	**0.01**	0.44	0.23	0.82				
Central nervous system	**0.038**	1.78	1.03	3.08				
Vascular	0.37	0.73	0.37	1.45				
HLA-B51	**5.4 × 10** ^**-4**^	2.21	1.41	3.46	**4.5 × 10** ^**-4**^	2.27	1.44	3.59

Forward stepwise logistic regression analysis was performed. Gastrointestinal, central nervous system, and vascular Behçet’s disease were not selected in the multivariate model

Change of treatment strategies, especially use of biologic agents, during the last two decades could have profound effects on the phenotypes of BD, as azathioprine has been suggested to modify the natural clinical course [17]. In Japan, the approval of infliximab (IFX) for uveitis in 2007, and adalimumab (ADA) for the GI type in 2013, have encouraged us to introduce biologic agents as early as possible in serious cases. Early initiation of biologic agents could suppress the appearance of additional symptoms, leading to phenotypical changes in recently enrolled patients in our cohort. We asked whether patients with IFX or ADA had different phenotypes among groups A, B, and C, but there were no significant differences among the groups in the rates of patients with the complete or GI type who were receiving TNF inhibitors, in spite of the increasing number of users (Additional file 2: Table S2). Patients who required anti-TNF therapy had already been found to have a mature BD phenotype at the start of therapy.

It is also possible that earlier detection of BD may lead to early intervention, resulting in a milder disease phenotype. However, there was no difference among the groups in the age of disease onset. Moreover, we have collected data on the use of prednisolone, colchicine, azathioprine, calcineurin inhibitors, methotrexate, and non-steroidal anti-inflammatory drugs (NSAIDs) in 456 patients for whom treatment data were available. As shown in Additional file 3: Table S3, we found that use of calcineurin inhibitors was decreased, while other drugs were increased. The results suggest that change of treatment strategy could have affected the BD phenotype in our patients. These data collectively indicate that the phenotype of BD is evolving in patients with recent onset of BD in our district.

## Discussion

In the present study, we observed a continuous reduction in complete-type BD in our district since the previous report in 2007 [8]. Moreover, we a reduced rate of genital ulcers, reduction in HLA-B51-positive patients, and increased prevalence of the GI type have persisted. Of these, HLA-B51 was found to be associated with the complete type in multivariate analysis. Therefore, it is likely that the decreased proportion of HLA-B51-positive patients is partially responsible for the recent phenotypical change, though a trend towards decline in the complete type was also observed in HLA-B51-positive patients. Although increment of the GI type, which was negatively associated with the complete type, was one of the important consequences in this study, it did not directly result in reduction in the complete type in our analysis. Of note, the analysis confirmed that the complete type was associated with an earlier date of BD onset. A similar evolution of the BD phenotype has been reported, not only in the Yokohama area, but also in other parts of Japan [7, 18], and recently in Korea [9]. Please change the ref3 to ref9 These data suggest that change in the BD phenotype is ongoing in East Asia.

The reduction in HLA-B51-positive BD patients in our cohort is intriguing. This phenomenon is difficult to explain, because the pathogenic role of HLA-B51 in the development of BD is not fully understood. Rather, our data suggest that interaction between HLA-B51-restricted immune responses and external factors is involved in the disease process, and that altered environmental factors attenuate the immune responses, resulting in reduced prevalence and phenotypical changes to mild forms in HLA-B51-positive individuals. Besides detailed molecular analysis, clinical observations as in this study may also contribute to understanding the pathogenesis of BD.

Several lines of evidence suggest that both genetic and environmental factors are involved in the pathogenesis of BD. An epidemiological study of immigration supports the hypothesis that BD has a primarily hereditary basis [19], while others report a significant impact of environmental factors, including life style, on disease development [20, 21]. Ethnic and regional differences in the clinical phenotypes of BD also suggest genetic factors are implicated in the clinical course after disease onset [22]. However, genetic backgrounds were highly preserved in a series of the cohort studies, because we have evaluated mostly Japanese patients. This was the case for other studies in Japan and Korea. Thus, it is likely that environmental factors contribute more to the phenotypical changes of BD in this area than genetic factors.

As for environmental factors, microbial pathogens are implicated in the development of BD [23]. Previously, we raised the possibility that improved oral hygiene contributes to the suppression of microbial pathogen-related immune responses [8]. It has long been suggested that microorganisms such as *Streptococcus sanguinis* provoke inflammation in BD [2, 23–25]. Smoking and gingivitis have been shown to be environmental factors, which potentially affect the components of oral flora [26]. Interestingly, both factors are also identified as predisposing factors in the development of rheumatoid arthritis through the generation of citrullinated peptides as autoantigens [27], though it seems unlikely that the same molecular mechanism operates in BD. Unfortunately, details of smoking were unavailable in this study.

BD flares are often caused by bacterial infection and after dental care, also suggesting oral flora involvement in the BD process [28, 29]. Moreover, our recent genetic association study implicated innate immune receptors involved in detecting microorganisms in the development of BD [4]. It is possible that change in environmental factors, such as resident microbial components, are altering functions of BD-susceptibility genes. However, which microorganisms, if any, are responsible for inflammation or reduction in the complete type in BD is unknown, and there is no evidence showing such microbiome structural alteration is actually occurring in Japan.

The present study showed that the factor most strongly attributing to reduction in complete type BD was genital ulcers, especially in female patients. Because it is likely that the decrease in prevalence of genital ulcers is caused by environmental factors, identification of the causative factors would give us a good clue in understanding the pathogenesis of BD.

The ISG criteria are one of the most commonly applied in BD studies [11]. The ISG criteria and the Japanese criteria use different sets of clinical information; the ISG criteria do not include clinical manifestations, which are listed as special types in the Japanese criteria, whereas the pathergy test is not included in the Japanese criteria. In spite of the discrepancy, the two previous independent genome-wide association studies (GWAS) have identified similar genetic architecture between Turkish BD (diagnosed using the ISG criteria) and Japanese BD (diagnosed using the Japanese criteria), suggesting that patients diagnosed with the two different sets of criteria are comparable [30, 31]. Indeed, the major conclusions of this study are not altered or limited in patients fulfilling the ISG criteria.

Consistent with previous studies, we found that the GI type was the only manifestation that increased among the serious phenotypes. The most apparent difference between the Japanese criteria and the ISG criteria lies in the presence of the GI type. Our previous paper reported that Japanese patients with GI-type BD fulfilled the ISG criteria at a lower rate compared with those with the non-GI type (with the GI type 70 % vs. 92 % without the GI type) [16]. It is still under consideration as to how to classify the GI type BD in patients who have a typical deep ulcer in the ileocecal region, but do not fulfill the ISG criteria. Our data suggest that due to increment of the GI type in East Asia, such patients should be followed carefully, as extensively discussed in a report from Korea [32].

It has long been recognized that the incidence of inflammatory bowel disease (IBD) such as Crohn’s disease is rising in many countries, and may be attributed to change to a high-fat diet [33]. In Japan, the prevalence of Crohn’s disease increased from 2.9 per 100,000 persons in 1986 to 63.6 in 2005 [34]. Similar mechanisms might be involved in the increased incidence of the GI type of BD. Indeed, *IL23R* and *IL10* have been identified in GWAS of BD [30], which are well-established IBD loci, supporting the notion that common mechanisms play roles in the development of GI-type BD.

Increment of GI involvement was associated with increased use of NSAIDs, suggesting that NSAIDs may be a risk factor for GI involvement in BD and in IBD. However, as significant association has been identified between GI involvement and arthritis [16], NSAIDs might be more frequently used in patients with GI-type BD because of complicated arthritis. Thus, it is hard to make conclusions about the cause-effect relationship between NSAIDs and GI involvement in the present study.

In our cohort, chronological changes in therapy could have affected the phenotypes and clinical course of disease. However, care should be taken to interpret the data because of possible bias caused by incomplete data collection, particularly in the early days. Moreover, the clinical diversity of BD makes it difficult to assess the disease-modifying effect of each therapeutic agent. It is hard to determine whether a particular symptom does not appear as a natural course or whether it has been suppressed by the preceding therapy. Moreover, because most treatments were determined by the attending physicians, the therapeutic scenario was not consistent in patients having a particular symptom, even on the same date.

Although we were unable to show significant effects of biologic treatment on the evolution of the BD phenotype, it is possible that immunosuppressive treatment prevented subsequent development of the complete-type and special-type BD. In a placebo-controlled trial of azathioprine, vascular involvement developed more frequently in the placebo group [17]. In addition, a case of vascular BD after termination of IFX was reported [35]. However, in the current study, we did not observe a significant reduction in eye disease, vascular type, or CNS involvement in BD; rather, there was increment of GI BD (Table 3). Patients with eye disease and special-type BD often require intensive immunosuppressive treatments, but not for genital ulcers (usually treated by topical or oral steroids). Because genital ulcers usually precede special-type manifestations [8], it is unlikely that recent therapeutic changes caused a reduction in genital ulcers during recent decades. However, due to the retrospective nature of our study, we cannot exclude the possibility of treatment effect on the evolution of the BD phenotype. To address the question, future prospective studies with larger sample size and longer observation period are needed.

There are several limitations in the study. First, the data were retrospectively collected, therefore were subject to selection bias. Second, data on important epidemiological factors such as smoking status, changes in diet, and body mass index were not available in all the patients. Third, because treatments were principally determined by the individual attending physicians and were not necessarily consistent among the patients, it was hard to assess the influence of therapeutic regimens on disease manifestations.

## Conclusions

Our cohort study showed that a reduced rate of complete-type and increased rate of GI-type BD are continuous trends in East Asia. Because the genetic background was consistently homogeneous in our study population, it is likely that the time-dependent changes were caused by environmental factors. Identification of causative factors would give a clue in the establishment of novel strategies to modify the clinical course and suppress disease flares as prophylactic medicine.

## Additional files

Additional file 1: Table S1. Phenotypes of patients with special-type BD. (DOC 32 kb) Additional file 2: Table S2. Phenotype of patients with BD treated with biologic agents. (DOC 29 kb) Additional file 3: Table S3. Rate of therapeutic agents use in groups with different date of onset. (DOC 31 kb)

## Abbreviations

ADA: adalimumab
BD: Behçet’s disease
CNS: central nervous system
GI: gastrointestinal
GWAS: genome-wide association studies
IBD: inflammatory bowel disease
IFX: infliximab
ISG: International Study Group
NSAIDs: non-steroidal anti-inflammatory drugs
TNF: tumor necrosis factor

## References

[1] Dalvi SR, Yildirim R, Yazici Y (2012). Behcet's Syndrome. Drugs.

[2] Isogai E, Ohno S, Kotake S, Isogai H, Tsurumizu T, Fujii N, Yokota K, Syuto B, Yamaguchi M, Matsuda H (1990). Chemiluminescence of neutrophils from patients with Behcet's disease and its correlation with an increased proportion of uncommon serotypes of Streptococcus sanguis in the oral flora. Arch Oral Biol.

[3] Sohn S, Lee ES, Bang D, Lee S (1998). Behcet's disease-like symptoms induced by the Herpes simplex virus in ICR mice. Eur J Dermatol.

[4] Kirino Y, Zhou Q, Ishigatsubo Y, Mizuki N, Tugal-Tutkun I, Seyahi E, Ozyazgan Y, Ugurlu S, Erer B, Abaci N (2013). Targeted resequencing implicates the familial Mediterranean fever gene MEFV and the toll-like receptor 4 gene TLR4 in Behcet disease. Proc Natl Acad Sci U S A.

[5] Round JL, Mazmanian SK (2009). The gut microbiota shapes intestinal immune responses during health and disease. Nat Rev Immunol.

[6] Consolandi C, Turroni S, Emmi G, Severgnini M, Fiori J, Peano C, Biagi E, Grassi A, Rampelli S, Silvestri E (2015). Behcet's syndrome patients exhibit specific microbiome signature. Autoimmun Rev.

[7] Wakabayashi T, Morimura Y, Miyamoto Y, Okada AA (2003). Changing patterns of intraocular inflammatory disease in Japan. Ocul Immunol Inflamm.

[8] Ideguchi H, Suda A, Takeno M, Ueda A, Ohno S, Ishigatsubo Y. Behcet disease: evolution of clinical manifestations. Medicine (Baltimore). 2011;90(2):125-132. 10.1097/MD.0b013e318211bf28 21358436

[9] Kim DY, Choi MJ, Cho S, Kim DW, Bang D (2014). Changing clinical expression of Behcet disease in Korea during three decades (1983-2012): chronological analysis of 3674 hospital-based patients. Br J Dermatol.

[10] Mizushima Y, Inaba G, Mimura Y (1987). Guide for the diagnosis of Behçet’s disease.

[11] Criteria for diagnosis of Behcet's disease. International Study Group for Behcet's Disease. Lancet. 1990;335(8697):1078–80. 1970380

[12] Saadoun D, Wechsler B, Desseaux K, Le Thi HD, Amoura Z, Resche-Rigon M, Cacoub P (2010). Mortality in Behcet's disease. Arthritis Rheum.

[13] Bonitsis NG, Luong Nguyen LB, LaValley MP, Papoutsis N, Altenburg A, Kotter I, Micheli C, Maldini C, Mahr A, Zouboulis CC (2015). Gender-specific differences in Adamantiades-Behcet's disease manifestations: an analysis of the German registry and meta-analysis of data from the literature. Rheumatology (Oxford).

[14] Maldini C, Lavalley MP, Cheminant M, de Menthon M, Mahr A (2012). Relationships of HLA-B51 or B5 genotype with Behcet's disease clinical characteristics: systematic review and meta-analyses of observational studies. Rheumatology (Oxford).

[15] Ishigatsubo Y, Takeno M, Ishigatsubo Y (2015). Overview. Behçet's Disease: From Genetics to Therapies.

[16] Ideguchi H, Suda A, Takeno M, Miyagi R, Ueda A, Ohno S, Ishigatsubo Y (2014). Gastrointestinal manifestations of Behcet's disease in Japan: a study of 43 patients. Rheumatol Int.

[17] Hamuryudan V, Ozyazgan Y, Hizli N, Mat C, Yurdakul S, Tuzun Y, Senocak M, Yazici H (1997). Azathioprine in Behcet's syndrome: effects on long-term prognosis. Arthritis Rheum.

[18] Kitamei H, Kitaichi N, Namba K, Kotake S, Goda C, Kitamura M, Miyazaki A, Ohno S (2009). Clinical features of intraocular inflammation in Hokkaido, Japan. Acta Ophthalmol.

[19] Mahr A, Belarbi L, Wechsler B, Jeanneret D, Dhote R, Fain O, Lhote F, Ramanoelina J, Coste J, Guillevin L (2008). Population-based prevalence study of Behcet's disease: differences by ethnic origin and low variation by age at immigration. Arthritis Rheum.

[20] Hirohata T, Kuratsune M, Nomura A, Jimi S (1975). Prevalence of Behcet's syndrome in Hawaii with particular reference to the comparison of the Japanese in Hawaii and Japan. Hawaii Med J.

[21] Zouboulis CC, Kotter I, Djawari D, Kirch W, Kohl PK, Ochsendorf FR, Keitel W, Stadler R, Wollina U, Proksch E (1997). Epidemiological features of Adamantiades-Behcet's disease in Germany and in Europe. Yonsei Med J.

[22] Lewis KA, Graham EM, Stanford MR (2007). Systematic review of ethnic variation in the phenotype of Behcet's disease. Scand J Rheumatol.

[23] Mumcu G, Inanc N, Yavuz S, Direskeneli H (2007). The role of infectious agents in the pathogenesis, clinical manifestations and treatment strategies in Behcet's disease. Clin Exp Rheumatol.

[24] Behcet H, Matteson EL (2010). On relapsing, aphthous ulcers of the mouth, eye and genitalia caused by a virus. Clin Exp Rheumatol.

[25] Lehner T, Lavery E, Smith R, van der Zee R, Mizushima Y, Shinnick T (1991). Association between the 65-kilodalton heat shock protein, Streptococcus sanguis, and the corresponding antibodies in Behcet's syndrome. Infect Immun.

[26] Rizvi SW, McGrath H (2001). The therapeutic effect of cigarette smoking on oral/genital aphthosis and other manifestations of Behcet's disease. Clin Exp Rheumatol.

[27] Catrina AI, Joshua V, Klareskog L, Malmstrom V (2016). Mechanisms involved in triggering rheumatoid arthritis. Immunol Rev.

[28] Mizushima Y, Matsuda T, Hoshi K, Ohno S (1988). Induction of Behcet's disease symptoms after dental treatment and streptococcal antigen skin test. J Rheumatol.

[29] Choi SM, Choi YJ, Kim JT, Lee SH, Park MS, Kim BC, Kim MK, Cho KH (2010). A case of recurrent neuro-Behcet's disease after tooth extraction. J Korean Med Sci.

[30] Remmers EF, Cosan F, Kirino Y, Ombrello MJ, Abaci N, Satorius C, Le JM, Yang B, Korman BD, Cakiris A (2010). Genome-wide association study identifies variants in the MHC class I, IL10, and IL23R-IL12RB2 regions associated with Behcet's disease. Nat Genet.

[31] Mizuki N, Meguro A, Ota M, Ohno S, Shiota T, Kawagoe T, Ito N, Kera J, Okada E, Yatsu K (2010). Genome-wide association studies identify IL23R-IL12RB2 and IL10 as Behcet's disease susceptibility loci. Nat Genet.

[32] Cheon JH, Kim ES, Shin SJ, Kim TI, Lee KM, Kim SW, Kim JS, Kim YS, Choi CH, Ye BD (2009). Development and validation of novel diagnostic criteria for intestinal Behcet's disease in Korean patients with ileocolonic ulcers. Am J Gastroenterol.

[33] Shoda R, Matsueda K, Yamato S, Umeda N (1996). Epidemiologic analysis of Crohn disease in Japan: increased dietary intake of n-6 polyunsaturated fatty acids and animal protein relates to the increased incidence of Crohn disease in Japan. Am J Clin Nutr.

[34] Asakura K, Nishiwaki Y, Inoue N, Hibi T, Watanabe M, Takebayashi T (2009). Prevalence of ulcerative colitis and Crohn's disease in Japan. J Gastroenterol.

[35] Magro-Checa C, Salvatierra J, Rosales-Alexander JL, Orgaz-Molina J, Raya-Alvarez E (2013). Life-threatening vasculo-Behcet following discontinuation of infliximab after three years of complete remission. Clin Exp Rheumatol.

