# Supplementary material for: Continuous evolution of clinical phenotype in 578 Japanese patients with Behçet’s disease: a retrospective observational study
Source: Arthritis Res Ther. 2016 Oct 3;18:217. doi: 10.1186/s13075-016-1115-x (PMC5048408; doi:10.1186/s13075-016-1115-x)
Supplement: Additional file 1: Table S1. — Phenotypes of patients with special-type BD. (DOC 32 kb) [file 13075_2016_1115_MOESM1_ESM.doc]

**Additional file 1: Table S1 Phenotypes of special type BD patients**

|  | **Non-special**  **n=419** | **GI**  **n=71*** | **CNS**  **n=59*** | **Vascular**  **n=46*** |
| --- | --- | --- | --- | --- |
| **Age at onset** | 37.4±12.2 | 33.6±13.1 | 35.5±9.9 | 36.6±12.6 |
| **Gender male (%)** | 172 (41.1) | 26 (36.6) | 34 (57.6) | 26 (44.1) |
| **HLA-B51 (%)** | 134/263 (51.0) | 12/33 (36.3) | 22/39 (56.4) | 14/30 (46.7) |
| **Complete type (%)** | 139 (33.2) | 13 (18.3) | 26 (44.1) | 12 (26.1) |
| **Oral ulcer (%)** | 414 (98.8) | 70 (98.6) | 59 (100) | 46 (100) |
| **Genital ulcer (%)** | 307 (73.3) | 52 (73.2) | 40 (67.8) | 33 (71.7) |
| **Eye involvement (%)** | 276 (65.9) | 21 (29.6) | 45 (76.3) | 19 (41.3) |
| **Skin involvement (%)** | 375 (89.5) | 58 (81.7) | 51 (86.4) | 43 (93.5) |
| **Complete type fulfilling ISG (%)** | 394 (94.0) | 47 (66.2) | 51 (86.4) | 37 (80.4) |

GI: gastrointestinal, CNS: central nervous system. *Some of the special type BD patients overlaps with other subtypes (e.g GI and vascular).
