# Supplementary material for: Continuous evolution of clinical phenotype in 578 Japanese patients with Behçet’s disease: a retrospective observational study
Source: Arthritis Res Ther. 2016 Oct 3;18:217. doi: 10.1186/s13075-016-1115-x (PMC5048408; doi:10.1186/s13075-016-1115-x)
Supplement: Additional file 2: Table S2. — Phenotype of patients with BD treated with biologic agents. (DOC 29 kb) [file 13075_2016_1115_MOESM2_ESM.doc]

**Additional file 2: Table S2 Phenotype of BD patients treated with biologics**

| **IFX/ADA** | **-2000** | **2000-2007** | **2008-** | ***p*** |
| --- | --- | --- | --- | --- |
| Observation time | 4.50±0.0 | 4.06±1.1 | 3.8±3.7 | NS |
| n (%, uncorrected) | 16/323 (5.0) | 20/164 (12.2) | 27/91 (29.729.6) | <0.0001 |
| n (%, corrected, <4.5 years) | 0/293 (0) | 11/160 (6.9) | 27/91 (29.7) | <0.0001 |
| Complete type (%) * | 6 (37.5) | 5 (25.0) | 9 (33.3) | NS |
| Gastrointestinal involvement (%) * | 1 (6.3) | 3 (15.0) | 3 (11.0) | NS |

IFX; infliximab, ADA; adalimumab, NS; not significant, *Analysis within the biologics-treated patients.
