# Supplementary material for: Continuous evolution of clinical phenotype in 578 Japanese patients with Behçet’s disease: a retrospective observational study
Source: Arthritis Res Ther. 2016 Oct 3;18:217. doi: 10.1186/s13075-016-1115-x (PMC5048408; doi:10.1186/s13075-016-1115-x)
Supplement: Additional file 3: Table S3. — Rate of therapeutic agents use in groups with different date of onset. (DOC 31 kb) [file 13075_2016_1115_MOESM3_ESM.doc]

**Additional file 3: TableS3 Use rate of therapeutic agents in the different onset date groups**

|  | **-2000**  n=233 | **2000-2007**  n=137 | **2008-**  n=86 | ***p*** |
| --- | --- | --- | --- | --- |
| Prednisolone (%) | 70(30.0) | 52 (38.0) | 36 (42.4) | 0.031 |
| Calcineurin inhibitors (%) | 59 (25.3) | 20 (14.6) | 6 (7.0) | <0.0001 |
| Azathioprine (%) | 3 (1.3) | 4 (2.9) | 7 (8.1) | 0.003 |
| Colchicine (%) | 106 (45.5) | 90 (65.7) | 77 (89.5) | <0.0001 |
| Methotrexate (%) | 3 (1.3) | 4 (2.9) | 6 (7.0) | 0.009 |
| NSAIDs (%) | 60 (25.8) | 55 (40.1) | 50 (58.1) | <0.0001 |
| TNF inhibitors (%)* | 0 (0) | 11 (6.9) | 27 (29.7) | <0.0001 |

NSAIDs; non-steroidal anti-inflammatory drugs.

* Total subjects were 293, 160, and 91 in -2000, 2000-2007, and 2008-, respectively.
